# S1 gene sequence analysis of a nephropathogenic strain of avian infectious bronchitis virus in Egypt

**DOI:** 10.1186/1743-422X-3-78

**Published:** 2006-09-20

**Authors:** Ahmed S Abdel-Moneim, Magdy F El-Kady, Brian S Ladman, Jack Gelb

**Affiliations:** 1 Department of Virology, Faculty of Veterinary Medicine, Beni-Suef University, Beni-Suef 62511, Egypt; 2 Department of Poultry Diseases, Faculty of Veterinary Medicine, Beni-Suef University, Beni-Suef 62511, Egypt; 3 Department of Animal and Food Sciences, University of Delaware, Newark, DE 19717, USA

## Abstract

**Background:**

Infectious bronchitis is highly contagious and constitutes one of the most common and difficult poultry diseases to control. IBV is endemic in probably all countries that raise chickens. It exists as dozens of serotypes/genotypes. Only a few amino acid differences in the S1 protein of vaccine and challenge strains of IBV may result in poor protection. Tropism of IBV includes the respiratory tract tissues, proventriculus and caecal tonsils of the alimentary tract, the oviduct and the kidney.

**Results:**

Infectious bronchitis virus (IBV) strain closely related to Massachusetts (Mass) serotype was isolated from broiler chickens suffering from severe renal and respiratory distresses. The isolate was serologically identified by Dot-ELISA and further characterized by RT-PCR then genotyped using S1 gene sequence analysis. Alignment of the S1 sequence of the isolate with 16 IBV strains revealed high homology to isolates related to Mass serotype. Inoculation with the strain reproduced the disease in experimental 1-day-old chickens and resulted in 20% mortality, severe renal and moderate respiratory distresses. Marked histopathological changes in both kidney and trachea were observed in experimentally infected chickens. A protection study using the H120 live attenuated vaccine showed low protection rate in spite of high S1 sequence homology (97%). Protection based criteria were: virus re-isolation attempts from trachea, tracheal and renal histopathology as well as IBV antigens detection by immunofluorescent antibody technique in kidney sections.

**Conclusion:**

Periodical evaluation of cross-protective capabilities of IBV vaccine(s) versus recently recovered field isolates should be performed to ensure optimum control of IBV.

## Background

Avian infectious bronchitis virus (IBV) is a highly contagious pathogen of chickens that replicates primarily in the respiratory tract and also in some epithelial cells of the gut, kidney and oviduct [1]. IBV is a virus member of genus Coronavirus, family Coronaviridae, order Nidovirales [2]. The virus possesses a positive stranded RNA genome that encodes phosphorylated nucleocapsid protein (N), membrane glycoprotein (M), spike glycoprotein (S) and small membrane protein (E). The spike glycoprotein is post-translationally cleaved into two subunits, S1 and S2 [1,3]. The S1 protein forms the N-terminal portion of the peplomer and contains antigenic epitopes mainly within three HVRs [4-6]. Neutralizing and serotype specific epitopes are associated within the defined HVRs [4,7,8].

Variation in S1 sequences [9-11], has been recently used for distinguishing between different IBV serotypes. Diversity in S1 probably results from mutation, recombination and strong positive selection in vivo [12]. Antigenically different serotypes and newly emerged variants from field chicken flocks sometimes cause vaccine breaks. The generation of genetic variants is thought to be resulted from few amino acid changes in the spike (S) glycoprotein of IBV [13,14].

In Egypt, isolates related to Massachusetts, D3128, D274, D-08880, 4/91 and the novel genotype; Egypt/Beni-Suef/01 were isolated from different poultry farms [15-18]. The commonly used IBV attenuated vaccine is H120 while the Mass 41 (M41) strain is commonly used in inactivated vaccines.

In the present study, Egypt/F/03 was isolated from 25-day-old broiler chickens in Fayoum Governorate, identified by Dot-ELISA, RT-PCR and sequenced to determine its serotype. Pathogenicity test to 1-day-old chickens and protection afforded by the commonly used H120 live attenuated vaccine were also performed.

## Results

### Virus isolation and serological identification

**Figure 1:**
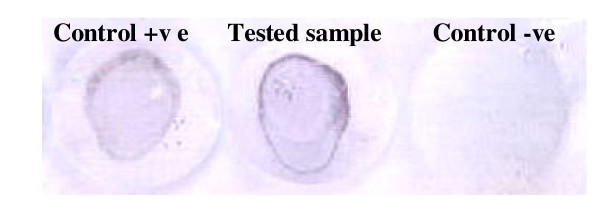
Dot-ELISA shows positive reaction in tested (chorioallantoic membrane homogenate) and control positive sample.

The allantoic fluid of the first chicken embryo passage of Egypt/F/03 was harvested at 48 h PI. Four additional egg passages were performed. Five eggs of the 4^th ^passage were incubated till being 18-day-old and all of them (100%) showed typical lesions of the IBV (stunting and dwarfing). The virus identity was ascertained by performing Dot-ELISA on the CAM homogenate (Fig. 1).

### Polymerase chain reaction and S1 gene cycle sequencing

**Figure 2:**
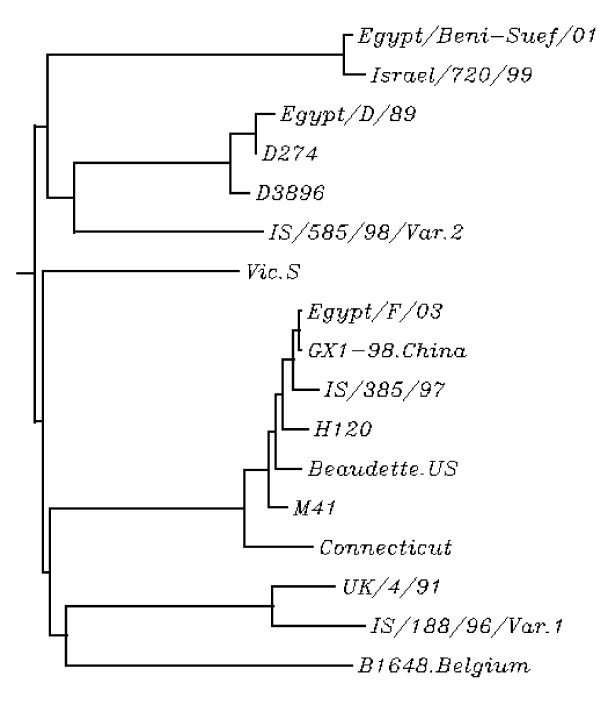
IBV S1 gene sequence relationships expressed as a phylogenetic tree of Egypt/F/03 isolate and selected IBV reference strains.

**Figure 3:**
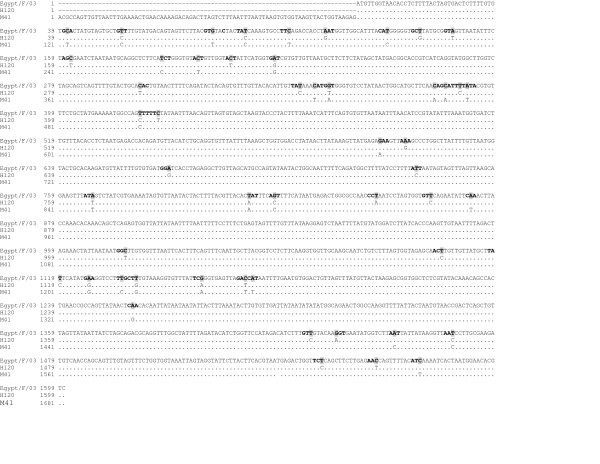
**Nucleotides identities of Egypt/F/03 with commonly used vaccine strains sequences**. Dots indicate residues identical to Egypt/F/03. Bold letters denotes codon areas. Shaded letters denote sites of differences.

**Figure 4:**
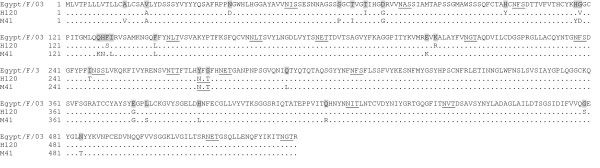
**Amino acid identities of Egypt/F/03 with commonly used vaccine strains sequences**. Dots indicate residues identical to Egypt/F/03. Potential glycosylation sites (NXS or NXT, except where X = P) are underlined. Shaded letters denote sites of differences. A:Alanine, C:Cysteine, D:Aspartic acid, E:Glutamic acid F:Pheny-lalanine, G:Glycine, H:Histidine, I:Isoleucine, K:Lysine, L:Leucine, M:Methionine, N:Asparagine, P:Proline, Q:Glutamine, R:Arginine, S:Serine, T:Threonine, V:Valine, W:Tryptophan, Y:tyrosine.

RT-PCR of Egypt/F/03 resulted in a product of 1600 base pairs using S1 primers OLIGO 5' and OLIGO 3'. Egypt/F/03 is closely related to the Beaudette US reference strain; 98% nucleotide identity and 96% amino acid identity (Table 1 and Fig. 2). It showed 97% similarities both in nucleotides and amino acids to H120 and 98% nucleotide and 96% amino acid homology to M41(Table 1). Egypt/F/03 showed 34 point mutations from H120; 20 silent and 14 non silent mutations. On the other hand, it showed 30 point mutations; 10 silent and 20 non silent mutations from M41 (Fig. 3, 4). Sixteen potential glycosylation sites were found in Egypt/F/03 while 17 were found in H120 and M41 (Fig. 4). All potential glycosylation sites found in Egypt/F/03 were shared with those found in H120 and M41(Fig. 4).

**Table 1:** Nucleotide and amino acid identities of Egypt/F/03 with selected IBV sequences

Nucleotide identity (%)																			
	**1**	**2**	**3**	**4**	**5**	**6**	**7**	**8**	**9**	**10**	**11**	**12**	**13**	**14**	**15**	**16**	**17**		
**1**		74	73	97	98	98	99	76	95	75	68	72	77	74	76	98	78	**1**	**Egypt/F/03**
**2**	71		73	74	74	74	75	74	73	76	76	77	75	98	73	75	79	**2**	**Egypt/Beni-Suef/01**
**3**	74	74		73	77	73	74	72	71	99	97	82	70	76	76	78	84	**3**	**Egypt/D/89**
**4**	97	71	73		97	97	97	76	94	75	68	73	77	73	75	97	78	**4**	**H120 **
**5**	96	70	72	96		97	94	76	95	79	75	81	77	73	75	96	77	**5**	**M41**
**6**	96	71	73	96	95		98	76	94	75	68	73	77	73	75	97	78	**6**	**Beaudette.US**
**7**	99	71	74	97	96	96		76	95	74	69	73	78	75	76	98	79	**7**	**GX1-98.China**
**8**	70	69	75	70	69	71	71		75	74	69	73	78	74	73	75	78	**8**	**B1648.Belgium **
**9**	90	70	72	91	90	89	90	68		73	67	72	76	72	73	91	76	**9**	**Connecticut **
**10**	74	75	98	74	72	74	74	75	73		91	82	73	76	76	78	84	**10**	**D274**
**11**	74	75	94	73	72	73	74	76	74	94		83	67	76	76	78	84	**11**	**D3896**
**12**	72	73	81	72	71	72	72	77	70	82	83		70	76	76	79	81	**12**	**Vic.S**
**13**	72	72	72	71	70	71	72	71	69	73	73	72		75	93	77	76	**13**	**UK/4/91**
**14**	68	97	73	68	68	68	69	66	67	73	73	69	71		73	74	79	**14**	**Israel/720/99**
**15**	71	68	72	69	68	70	71	69	67	73	73	71	88	69		76	76	**15**	**IS/188/96/Var.1**
**16**	97	71	73	96	93	96	97	70	84	73	73	72	72	70	71		78	**16**	**IS/385/97**
**17**	71	77	83	71	70	71	72	76	69	83	85	77	73	78	73	72		**17**	**IS/585/98/Var.2**
	**1**	**2**	**3**	**4**	**5**	**6**	**7**	**8**	**9**	**10**	**11**	**12**	**13**	**14**	**15**	**16**	**17**		

Amino acid identity (%)																			

### Virulence test

**Figure 5:**
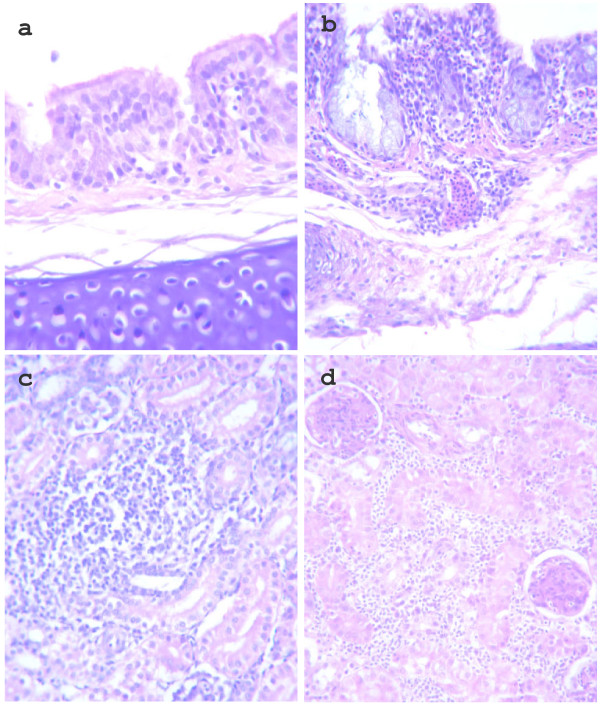
**Trachea and kidney histopathology following experimental infection of 1-day old chickens with Egypt/F/03**. Trachea and kidney stained with H & E: a. Trachea of chickens 5 d P.I with Egypt/F/03 showed hyperplasia, lymphcytic infiltration and oedema (40 ×). b. Trachea of chickens 7 d P.I with Egypt/F/03 showed diffuse lymphocytic aggregation, degeneration of the epithelium mucus, and haemorrhages (20 ×). c. Kidney of chickens 5 d P.I with Egypt/F/03 showed focal lymphocytic aggregation in the interstitium and in the glomeruli, as well as degenerative changes in tubular epithelium (40 ×). d. Kidney of chickens 7 d P.I with Egypt/F/03 showed massive renal haemorrhages and degeneration renal tubular epithelium (20 ×).

Chickens inoculated with Egypt/F/03 exhibited snicking and rales in approximately 50% of infected birds at 3^rd ^day of inoculation. Conjunctivitis was observed in 20/30 at 3^rd ^day PI that was elevated to 22/30 by the 5^th ^day PI of infected birds whereas no birds exhibited watery eyes. Six birds were dead after Egypt/F/03 experimental infection; 4 birds in the 7^th ^day PI and 2 birds in the 8^th ^day PI. Post-mortem examination of dead birds, revealed petechial haemorrhages in larynx and thymus, severe congestion of liver, spleen and lungs as well as renal haemorrhages. These changes appeared but in milder form in birds sacrificed at 5 days PI. Histopathological examination of sacrificed birds at 5 days PI and freshly dead birds at 7 days PI revealed mucus, marked loss of cilia, desquamation, mononuclear infiltration, epithelial hyperplasia and vascular congestion of the trachea (Fig. 5). Kidneys showed severe changes including haemorrhages, degenerative changes in renal tubules and hypercellularity of the renal glomeruli as well as focal lymphocytic infiltration (Fig. 5). In general the tracheal and renal histopathological lesions were more severe in dead birds (Fig. 5b, d) than birds sacrificed at 5 days PI (Fig. 5a, c).

### Vaccination trial

Chickens vaccinated with H120 (Group A) showed 58.3% protection (7/12) by virus reisolation procedure and 66.6% (8/12) protection by histopathology after challenge with Egypt\F/03 while all control unvaccinated birds (Group B) were not protected (5/5) (Table 2). On day four PI, the kidneys of (3/12) birds in group A (vaccinated and challenged with IBV) showed focal lymphocytic infiltration, urates deposition and degenerative changes in renal tubules while birds of group B (unvaccinated and challenged with IBV) showed multifocal lymphocytic infiltration, urates deposition and degenerative changes in renal tubules in 4/5 birds, while only focal lymphocytic infiltration, urates deposition and degenerative changes in renal tubules were observed in 1/5 birds. Birds in group C (vaccinated unchallenged group) did not show any abnormalities (0/12) either in tracheae or kidneys. Immunofluorescenct assay on kidneys of challenged and unchallenged groups showed that all challenged unvaccinated and 3/12 of challenged vaccinated groups possessed kidney immunofluorescence. None of control unchallenged groups possessed kidney immunofluorescence (Table 2).

**Table 2:** Protection of chickens following vaccination with IBV H120 strain and challenged with Egypt/F/03

				Tracheal Protection				Kidney Protection						
				
Group^A^	n^B^	Vaccination	Challenge^C^	Virus Reisolation^D^		Tracheal Histopathology^E^		Kidney histopathology				Immunoflourscence		
						
				Positive	Negative	Positive	Negative	Normal	Focal	Multifocal	Diffuse	Positive	Negative	Intensity^F^
A	12	+	+	5	7	4	8	9	3	0	0	3	9	+
B	5	-	+	5	0	5	0	0	3	4	0	5	0	+++
C	12	+	-	0	12	0	12	12	0	0	0	0	12	-

^A^Groups A and C were vaccinated by IBV H120 vaccine at 1-day of age while birds in group B were kept as unvaccinated control.

^B^Number of birds used per group.

^C ^Four weeks post vaccination; chickens in group A and B were challenged by eye drop with 10^5 ^EID_50 _per bird of Egypt/F/03 while birds in group C were not challenged and kept as vaccinated unchallenged control:(-) denotes unchallenged, while (+) denotes challenged groups.

^D ^Virus reisolation 4 days post challenge with Egypt/F/03 in SPF ECE. Unprotected tracheal samples showed positive IBV reisolation while protected tracheal samples showed negative results.

^E^Tracheal histopathology 4 days post challenge with Egypt/F/03 was measured as tracheal histopathological scores. Tracheal samples showed high tracheal histopathological scores (score range: 6–8) were considered positive (unprotected). Negative (protected) tracheal scores range was 1–2.

^F ^Intensity of IFA: (+) denotes few cells showed positive IFA reaction, (++) many cells showed IFA reaction, (+++) diffuse IFA reaction.

## Discussion

In this study, an Egyptian IBV strain; Egypt\F/03 was isolated from a tissue pool of kidney and trachea from unvaccinated broiler flock with a history of respiratory and renal disease. The strain produced typical lesions of IBV in inoculated embryos and identified as IBV by Dot-ELISA and RT-PCR. The isolate was found to be devoid of major concomitant viruses; avian influenza virus, Newcastle disease virus, infectious laryngotracheitis virus, reovirus and adenovirus (data not shown).

S1 sequence analysis of Egypt/F/03 revealed its close relatedness to Mass serotype. It showed high nucleotide similarities to GX1-98.China (99% nucleotide and amino acid identities), Beaudette-US (98% nucleotide and 96% amino acid identities), IS/385/97 (98% nucleotide and 97% amino acid identities), H120 (97% nucleotide and amino acid identities) and M41 (98% nucleotide and 96% amino acid identities).

It is known that the most severe clinical response of IBV appears in very young chickens and severity is alleviated in older chickens [19,20]. This fact explains the high mortality rate observed in 1-day-old chickens that experimentally inoculated with Egypt/F/03 compared to mortality pattern in the original flock (25-day-old chickens). The presence of acute interstitial nephritis on days 5 and 7 post infection indicated that Egypt\F\03 is a nephrogenic IBV. The microscopic findings of the renal tubules matched the general findings recorded with nephrogenic IBV strains [21,22]. The microscopic findings in tracheal sections appeared similar to those recorded by [19,21] including: loss of cilia, degenerative changes of the tracheal mucosa, irregular loss of epithelium, desquamation of the sloughed epithelium in the tracheal lumen and lymphocytic infiltration that ranged from focal aggregation to diffuse massive infiltration. Severe renal haemorrhages observed grossly and in hisopathological sections of birds dead after experimental infection with Egypt/F/03 denote that deaths resulted from acute renal failure. Our finding regarding the presence of petechial haemorrhages in larynx and thymus as well as severe congestion of liver, spleen and lungs in birds dead after IBV experimental infection is in agreement with [23,24] who confirmed the presence of IBV viral antigens in such organs.

Evaluation of the immune response to IBV vaccination is based on several criteria including: clinical signs, tracheal histological lesion, virus neutralization, virus re-isolation from trachea, antigen detection in trachea and/or kidney by immunofluorescent or immunoperoxidase techniques [25-29]. In this study we used tracheal histological lesion and virus re-isolation as parameters for tracheal protection. Kidney histopathology and antigen detection by IFA were used as indicators of kidney protection. Complete protection is expected upon using closely related vaccine strain as the degree of cross protection among IBV strains generally reflects the similarities between the S proteins [12,30]. The re-isolation of Egypt/F/03 from the trachea of vaccinated birds and the presence of tracheal and renal microscopic lesions as well as viral antigen in kidneys (by IFA) in H120 vaccinated birds denote lack of complete protection afforded by H120 vaccination.

H120 is a mild vaccine and it is possible that the challenge virus was too virulent for the level of immunity that the vaccine produced in these young chickens. Other possible consideration includes that baby chickens are not fully immunocompetent at one-day of age, the time that they were vaccinated for the protection study experiment. However, commercial broiler chickens possess maternally derived antibodies, are routinely vaccinated at one-day of age [31,32] without apparent interference by the maternal derived antibodies in the development of active immunity, at least in the respiratory tract that measured by challenge [33]. On the other hand, variable results were recorded regarding homologous protection of IBV. Cavanagh et al. [12] inoculated groups of 10 chickens with the virulent UK/6/82 isolate and challenged with isolates that differed by up to 4% of S1 amino acids. Challenge with two variants (98% S1 identity with UK/6/82) resulted in challenge scores virtually the same as with the homologous challenge however, challenge with two others isolates (96% and 98% S1 identity, respectively), resulted in less cross-protection, although the numbers were not statistically significantly different.

In the S1 subunit, three HVRs are located within amino acids 38–67, 91–141 and 274–387 [4-6]. HVR1 and HVR2 contain sequences that have been associated with specific IBV serotypes [34,35] as well as serotype specific neutralizing epitopes [4,5,14]. IBV serotypes commonly differ by 20 to 25% in S1 [11,36] but some serotypes differ in S1 by as little as 2% [13]. Although H120 showed 97% amino acid and nucleotide identity to Egypt/F/03, it possesses 34 different nucleotides that resulted in 14 amino acid substitutions. Among such amino acids, one is located in HVR1, four in HVR2 and one in HVR3 (Fig.4). The region between amino acid residues 123–152 has been previously identified as a possible region involved in the differing pathogenicity of Gray and non-virulent JMK strains [37]. Egypt/F/03 possesses different amino acid; phenylalanine within this region at positions 130 and 141 instead of serine and leucine in H120 respectively. It is apt to mention that some serotypes differ in S1 by as little as 10 amino acids [13], suggesting that only a few epitopes may induce most of the VN antibody [38].

## Conclusion

Egypt/F/03 is a nephropathogenic IBV strain closely related to Mass serotype. Vaccination by H120 did not provide satisfactory protection against challenge with Egypt/F/03. Complete protection of trachea against the Egypt/F/03 and consequently efficient prevention of kidney infection may be quite feasible upon development of safe attenuated vaccines based on indigenous field strain. Preparation of live and inactivated vaccines from indigenous isolates should parallel periodic evaluation of cross-protective capabilities of such vaccine(s) versus recently recovered field isolates in order to ensure optimum control of IBV.

## Methods

### Embryonated chicken eggs

SPF ECE obtained from Nile SPF (Koom Oshiem, Fayoum, Egypt) were used for isolation of the field isolate, serial passages, titration of the seed stocks of Egypt/F/03 and vaccine strain (H120), as well as virus re-isolation attempts following challenge in the protection study.

### Chickens

Sixty nine commercial 1-day-old chickens (El-Waddi Co, Egypt) were reared under strict hygienic conditions in separate rooms and used in both virulence test and protection study.

### Rabbit anti-IBV

Rabbit anti-IBV polyclonal antiserum raised against vero adapted H120 vaccine was prepared previously in our lab [17] and used for detection of IBV antigens in both Dot-ELISA and indirect immunofluorescent antibody technique.

### Clinical history

Infectious bronchitis was diagnosed during Augest 2003 in Fayoum Governorate, Egypt. The outbreak occurred in 25-day-old commercial broiler farm with no previous IBV vaccination. The flock was vaccinated against Newcastle disease and infectious bursal disease viruses at 14 and 18 days of age respectively. The total flock density was 3000 birds. The first signs were depression and respiratory distresses including sneezing, coughing and rales. Other signs included conjunctivitis and watery eyes. Within a period of 10 days after the appearance of the disease, the mortality rate increased to 10% of the flock density. Post-mortem examination of dead birds revealed increased tracheal mucus, severe renal congestion, urates filled ureters as well as congestion in liver and spleen.

### Virus isolation and passage in SPF ECE

Egypt/F/03 was isolated from 25-day-old broiler chickens suffering from both respiratory and renal distresses from Fayoum Governorate in 2003. A kidney homogenate (10% in sterile PBS) and a tracheal scraping suspension were pooled, centrifuged at 500 × g for 10 min. The supernatant fluid was inoculated into chorioallantoic sac of 10-day-old SPF ECE. Allantoic fluid was harvested after 48 h and was used for re-passage into ECE. Five eggs of the 4^th ^egg passage were incubated till being 18-day-old and examined for typical lesions of IBV (stunting, curling and urates deposition in ureters).

### Dot-ELISA for virus identification

A Dot-ELISA was performed according to [39]. Briefly, NCM of convenient size was cut, marked with waterproof ink for identification and then soaked for 10 min. in distilled water. NCM was laid on absorbent paper and air-dried for 5 min. Three μl of CAM homogenate of the 4^th ^virus passage of Egypt/F/03, positive control (CAM homogenate 48 h after inoculation of H120 vaccine) and negative control (normal CAM homogenate) samples were applied as small spots on the membrane. The dotted membrane was allowed to air dry for 15 min then blocked for 30 min. in Tris buffer (20 mM Tris base, 500 mM NaCl pH 7.5) containing 0.5% Tween 20 then rinsed for 5 min. in Tris buffer. NCM was then incubated for 1 h with rabbit anti-IBV (prepared previously in our lab.) predilluted to 1:10 with diluents buffer (Tris buffer containing 0.05% Tween20). Bound antibodies were detected by incubating NCM for 1 h with goat anti-rabbit peroxidase conjugate (Sigma, Co.) prediluted to 1:500 with diluent buffer. NCM was rinsed three times (10 min. each) with Tris buffer after each step. Finally the membrane was incubated for 15 min. in 60 mm Petri dish containing 20 ml 4-chloro-1-naphthol and hydrogen peroxide substrate working solution. The membrane was rinsed with water to stop the enzymatic reaction. Blue dots denote positive reaction.

### Viral inactivation, polymerase chain reaction and S1 gene cycle sequencing

Egypt/F/03 was inactivated by treating 2 ml of the infective allantoic fluids with an equal volume of molecular biology grade phenol (pH 4.3) (Fisher Scientific, Fair Lawn, NJ). Following inactivation, the isolate was shipped to the University of Delaware as stipulated by an USDA Veterinary Import Permit issued to J. Gelb, Jr. The phenol-treated allantoic fluid was vortexed and then centrifuged at 12,000 × g for 3 min. The supernatant was harvested and an additional treatment using phenol/chloroform/isoamyl alcohol (pH 4.3) (Fisher Scientific) was performed. Viral RNA was harvested from the aqueous layer and extracted using a Qiagen Viral RNA Mini Kit (Qiagen, Inc., Valencia, CA). The RNA was eluted in sterile diethyl pyrocarbonate (DEPC)-treated water and stored at -70°C. RT was performed on the viral RNA using the GeneAmp RNA PCR Core Kit (Applied Biosystems, Foster City, CA). Approximately 2 μl of the extracted viral RNA was used to synthesize cDNA. Amplification of the S1 gene was performed using S1OLIGO3' (5'-CATAACTAACATAAGGGCAA-3') and S1OLIGO5' (5'-TGAAACTGAACAAAAGAC-3') primers [10,37]. PCR of S1 gene was performed as described [11] with the exception that extension was performed at 60°C.

S1 PCR product was cut from 1.8% agarose gels, purified with the QIAquick gel extraction kit (Qiagen, Inc.) and the DNA was quantitated as described [11]. Purified RT-PCR product was sequenced in the forward and reverse directions using the same primers. Sequencing was performed as described [11].

### Sequence analysis

A BLAST^® ^analysis [40] was initially performed using the S1 sequence of Egypt/F/03 (DQ487085) to establish its identity to GenBank accessions. A comparative analysis of S1 sequences was performed using the CLUSTAL W Multiple Sequence Alignment Program, version 1.83 [41]. The tree was constructed using the neighbour-joining program [41]. IBV S1 sequences representative to genotypes used for the alignments were obtained from the GenBank and EMBL database. They include: Egypt/Beni-Suef/01 (AF395531), Egypt/D/89 (DQ487086) H120(M21970), M41(M21883), Beaudette.US (AJ311362), GX1-98.China(AY319302), Connecticut(L18990), B1648.Belgium (X87238), D274 (X15832), D3896 (X52084), Vic.S (U29519), UK/4/91(AF093794), Israel/720/99(AY091552), IS/188/96/Var.1(AY789949), IS/385/97(AY789957) and IS/585/98/Var.2 (AY789962). Egypt/F/03 was compared with H120 and M41 vaccine sequences using multisequence alignment [42] and sequences were presented using BOXSHADE 3.21 [43].

### Virulence test

Forty 1-day-old chickens were used. Thirty chickens were infected by intraocular instillation of 10^5 ^EID_50_/100 μl of Egypt/F/03 according to [21] while other birds were kept as control uninfected group. Clinical signs and gross post-mortem lesions as well as mortalities were recorded. Microscopic examinations of both tracheae and kidneys were performed at 5 and 7 days post infection.

### Protection study

Twenty nine commercial 1-day-old chickens were used to evaluate the protection provided by H120 vaccination against challenge with Egypt/F/03. Birds were divided into three groups; A (n = 12), B (n = 5), C (n = 12). Vaccination was performed at day 1 by eye drop application. Single dose of H120 vaccine (Nobilis, Intervet, The Netherlands BV) was used for each bird in groups A and C according to manufacturer's instructions while birds in group B were kept as unvaccinated control. Four weeks post vaccination, chickens in group A and B were challenged by eye drop with Egypt/F/03 (10^5 ^EID_50 _per bird) while birds in group C were not challenged and kept as vaccinated unchallenged control. Tracheae of all birds from all groups were collected four days post challenge for virus reisolation attempts and histopathological examination. Tracheal scrapings were emulsified in 2 ml of sterile PBS and centrifuged at 500 × g for 3 min. Virus reisolation attempts were performed by inoculating 2–3, 10-day-old SPF ECE by the supernatant fluid of each sample as described [44]. Embryos were examined for typical lesions of IBV. For histopathological examination, tracheae were fixed in formalin, processed routinely for histopathology and stained with haematoxylin and eosin. The trachea from each bird was examined microscopically and assigned lesion scores of 0–3 with 0 = none, 1 = focal, 2 = multifocal, 3 = diffuse. Tracheae were scored for the amount of mucous, loss of cilia, epithelial hyperplasia, necrosis, lymphocyte and heterophil infiltrations as well as the extent of tissue reaction. The scores for each bird were added and the mean score for the birds in each group was calculated [45]. Kidney samples were also taken 4 days post challenge and examined microscopically for tubular degeneration and inflammation consistent with interstitial nephritis. Focal, multifocal and diffuse were used to assign kidney histopathology. The presence of viral antigens in kidneys was screened by immunofluorescent antibody technique.

### Indirect immunofluorescent antibody technique (IFA)

It was performed according to [46] to detect viral antigens in the kidneys of birds after challenge with Egypt/F/03 in protection study. Briefly, deparaffinized slides were incubated with rabbit anti-IBV antibodies (1:5) for 1 h and subsequently with FITC-conjugated goat anti-rabbit antibody (Kirkegaard and Perry Laboratories, Gaithersburg, Md.) (1:1000) for 1 h. Both primary and secondary antibodies were diluted in PBS. Slides were rinsed three times (10min./single wash) with PBS after each step. Slides were then mounted using glycerol/PBS (without allowing the slides to dry) and examined under fluorescent microscopy.

## Abbreviations

CAM, chorioallantoic membrane; ECE, Embryonated chicken eggs; EID, egg infective dose fifty; FITC, fluorescien isothiocynate; HVR, hypervariable region; IBV, infectious bronchitis virus; NCM, nitrocellulose membrane; PBS, phosphate buffer saline; RT, reverse transcriptase; SPF, specific-pathogen-free.

## Competing interests

The author(s) declare that they have no competing interests.

## Authors' contributions

ASA isolated and serologically characterized Egypt/F/03 virus and performed virulence test as well as protection study. He also performed multisequence alignment, phylogenetic analysis and drafted the manuscript, MFE provided sample for isolation, helped in performing virulence test and protection study and reviewed the manuscript, JGJr helped in performing RT-PCR, S1 gene sequence of Egypt/F/03 and critically reviewed the manuscript, BSL made RT-PCR and S1 gene sequence of Egypt/F/03.
